# EEG features in depressive female adolescents with suicidal and non-suicidal auto-aggressive behavior

**DOI:** 10.1192/j.eurpsy.2021.464

**Published:** 2021-08-13

**Authors:** E. Iznak, E. Damyanovich, I. Oleichik, N. Levchenko

**Affiliations:** 1 Laboratory Of Neurophysiology, Mental Health Research Centre, Moscow, Russian Federation; 2 Clinical Department Of Endogenous Mental Disorders And Affective States, Mental Health Research Centre, Moscow, Russian Federation

**Keywords:** female adolescents, non-suicidal self-injury, suicidal attempts, Depression

## Abstract

**Introduction:**

In adolescents, both non-suicidal self-injuries (NSSI) and previous suicidal attempts (SA) represent significant risk factors for future suicide. Thus, the search for EEG markers of these forms of auto-aggressive behavior seem to be an actual task.

**Objectives:**

The aim of the study was to reveal the differences of baseline EEG features in depressive female adolescents with auto-aggressive behavior such as NSSI or SA.

**Methods:**

The study included 45 depressive female in-patients aged 16–25 years. 21 of them showed only NSSI (NSSI subgroup), 24 patients had a history of SA (SA subgroup). Subgroups did not differ in clinical and social-demographic parameters. Baseline EEG spectral power (SP) and its asymmetry were measured.

**Results:**

SA subgroup had higher parietal-occipital alpha-2 (9-11 Hz) SP than NSSI subgroup. Its focus was located in the right hemisphere, and alpha-3 (11-13 Hz) SP was higher than alpha-1 (8-9 Hz). In contrary, in NSSI subgroup alpha-1 SP was higher than alpha-3; and foci of alpha-2 and alpha-3 SP were localized in the left hemisphere.

**Conclusions:**

Spatial distribution and the ratio of EEG alpha frequency components SP in the SA subgroup reflect greater activation of brain cortex, especially of the left hemisphere that is more typical for EEG of individuals with increased risk of suicide. In NSSI subgroup, the right hemisphere is relatively more activated that is more typical for EEG in depression without SA. The study supported by RBRF grant No.20-013-00129a.

**Disclosure:**

No significant relationships.

